# Role of Immune Dysregulation in Increased Mortality Among a Specific Subset of COVID-19 Patients and Immune-Enhancement Strategies for Combatting Through Nutritional Supplements

**DOI:** 10.3389/fimmu.2020.01548

**Published:** 2020-07-09

**Authors:** Kosagi-Sharaf Rao, Vaddi Suryaprakash, Rajappa Senthilkumar, Senthilkumar Preethy, Shojiro Katoh, Nobunao Ikewaki, Samuel J. K. Abraham

**Affiliations:** ^1^Centre for Neuroscience, Instituto de Investigaciones Científicas y Servicios de Alta Tecnología (INDICASAT AIP), Panama City, Panama; ^2^Department of Urology, Yashoda Hospitals, Hyderabad, India; ^3^The Fujio-Eiji Academic Terrain, Nichi-In Centre for Regenerative Medicine, Chennai, India; ^4^Edogawa Evolutionary Laboratory of Science, Edogawa Hospital, Tokyo, Japan; ^5^Department of Medical Life Science, Kyushu University of Health and Welfare, Nobeoka, Japan; ^6^Institute of Immunology, Junsei Educational Institute, Nobeoka, Japan; ^7^The Mary-Yoshio Translational Hexagon, Nichi-In Centre for Regenerative Medicine, Chennai, India; ^8^School of Medicine, Yamanashi University, Yamanashi, Japan; ^9^GN Corporation Co. Ltd., Kofu, Japan

**Keywords:** COVID-19, co-morbidity, diabetes, hypertension, chronic kidney disease, immuno-compromised, nutritional supplement, AFO-202 beta glucan

## Abstract

**Background:** The COVID-19 pandemic has been causing varying severities of illness. Some are asymptomatic and some develop severe disease leading to mortality across ages. This contrast triggered us explore the causes, with the background that a vaccine for effective immunization or a drug to tackle COVID-19 is not too close to reality. We have discussed strategies to combat COVID-19 through immune enhancement, using simple measures including nutritional supplements.

**Discussion:** A literature search on mortality-related comorbid conditions was performed. For those conditions, we analyzed the pro-inflammatory cytokines, which could cause the draining of the immune reservoir. We also analyzed the immune markers necessary for the defense mechanism/immune surveillance against COVID-19, especially through simple means including immune enhancing nutritional supplement consumption, and we suggest strategies to combat COVID-19. Major comorbid conditions associated with increased mortality include cardiovascular disease (CVD), diabetes, being immunocompromised by cancer, and severe kidney disease with a senile immune system. Consumption of *Aureobasidium pullulans* strain (AFO-202) beta 1,3-1,6 glucan supported enhanced IL-8, sFAS macrophage activity, and NK cells' cytotoxicity, which are major defense mechanisms against viral infection.

**Conclusion:** People with co-morbid conditions who are more prone to COVID-19-related deaths due to immune dysregulation are likely to benefit from consuming nutritional supplements that enhance the immune system. We recommend clinical studies to validate AFO-202 beta glucan in COVID-19 patients to prove its efficacy in overcoming a hyper-inflammation status, thus reducing the mortality, until a definite vaccine is made available.

## Introduction

The outbreak of the ongoing COVID-19 pandemic started at the end of 2019, in the city of Wuhan, China. COVID-19 has been attributed to a novel type of coronavirus, termed by the WHO as the “novel coronavirus-2019” (SARSCoV-2). The genome sequence of SARSCoV-2 is similar to those of severe acute respiratory syndrome coronavirus (SARS-CoV) (~79% homology), whose outbreak occurred in 2002 and 2003, and of Middle East respiratory syndrome coronavirus (MERS-CoV) (~50% homology), whose outbreak occurred between 2012 and 2019. Coronavirus is member of the family Coronaviridae and subfamily Coronavirinae, which, based on genomic sequencing and phylogenetic relationships, consists of four genera: Alphacoronavirus, Betacoronavirus, Gammacoronavirus, and Deltacoronavirus. SARS-CoV-2 belongs to the Betacoronavirus genus (1, 2).

As of 3 May 2020, there have been 3,356,205 confirmed cases of COVID-19, including 238,730 deaths, reported to the WHO (3). The incubation period of SARS-CoV-2 is 3–6 days, with the maximum being 14 days. The clinical signs and symptoms of COVID-19 include low to high fever, non-productive cough, myalgia, dyspnea, fatigue, standard or decreased leukocyte counts, and confirmed evidence of pneumonia on chest radiography. Less common symptoms of SARS-CoV-2 infection include headache, abdominal pain, dizziness, nausea, vomiting, and diarrhea. Regarding the therapeutic aspects, no specific therapy is currently available for COVID-19 (4). Patients with mild signs and symptoms are treated with antibacterial drugs for pneumonia, including azithromycin, fluoroquinolones, and amoxicillin. Anti-viral agents such as viral methyltransferase inhibitor, nitazoxanide, the nucleotide prodrug GS-5734 Remdesivir, ribavirin in combination with lopinavir, interferon therapy, and convalescent plasma therapy are being tested for treating COVID-19. The case fatality rate (CFR) was reported to be between 2.3% (1,023 deaths among 44,672 confirmed cases) in China up to 15.80% in the UK (4, 5). In particular, patients with co-morbid conditions are at higher risk of mortality from COVID-19 due to the conditions compromising their immune system (4). Here, we present the immune system implications of COVID-19 in the presence of co-morbidities and ways to enhance immunity, with a focus on nutritional supplements.

## COVID-19 and the Immune System

A characteristic feature of COVID-19 infection is a pro-inflammatory status characterized by high levels of different cytokines, including interleukin (IL)-1β, IL-1Rα, IL-2, IL-10, fibroblast growth factor (FGF), granulocyte-macrophage colony stimulating factor (GM-CSF), granulocyte-colony stimulating factor (G-CSF), interferon-γ-inducible protein (IP10), monocyte chemoattractant protein (MCP1), macrophage inflammatory protein 1 alpha (MIP1A), platelet-derived growth factor (PDGF), tumor necrosis factor (TNFα), and vascular endothelial growth factor (VEGF). Furthermore, critically ill patients requiring admission to the intensive care unit (ICU) were found to have markedly high concentrations of IL-2, IL-10, G-CSF, IP10, MCP1, MIP1A, TNFα, and IL-6. Importantly, increased levels of IL-6 are also correlated with increased mortality. In severe COVID-19, a reduction of natural killer cells—CD4+ and CD8+ T lymphocytes—and IFN-γ expression in CD4+ cells have been observed, along with hampered adaptive immune systems due to cytokine release syndrome, which can be attributed to the inverse correlation of levels of IL-6, IL-10, and TNFα with lymphocyte count (6–8). In another report, lymphopenia with drastically reduced numbers of CD4+ T cells, CD8+ T cells, B cells, and natural killer (NK) cells was reported to be a common feature in patients with severe COVID-19, which was not observed in milder cases. In addition, the numbers of CD4+ T cells, CD8+ T cells, B cells, and NK cells are normalized in patients who have recovered or are convalescent. The exhaustion markers, such as NKG2A, on cytotoxic lymphocytes, including NK cells and CD8+ T cells, were increased in those with severe disease and returned to normal levels after recovery from COVID-19 (9). Increased neutrophil-to-lymphocyte ratio (NLR) and low lymphocyte-to-C-reactive protein ratio (LCR), reflecting an enhanced inflammatory process, have been reported to suggest a poor prognosis in patients with severe COVID-19 (10).

**Figure 1:**
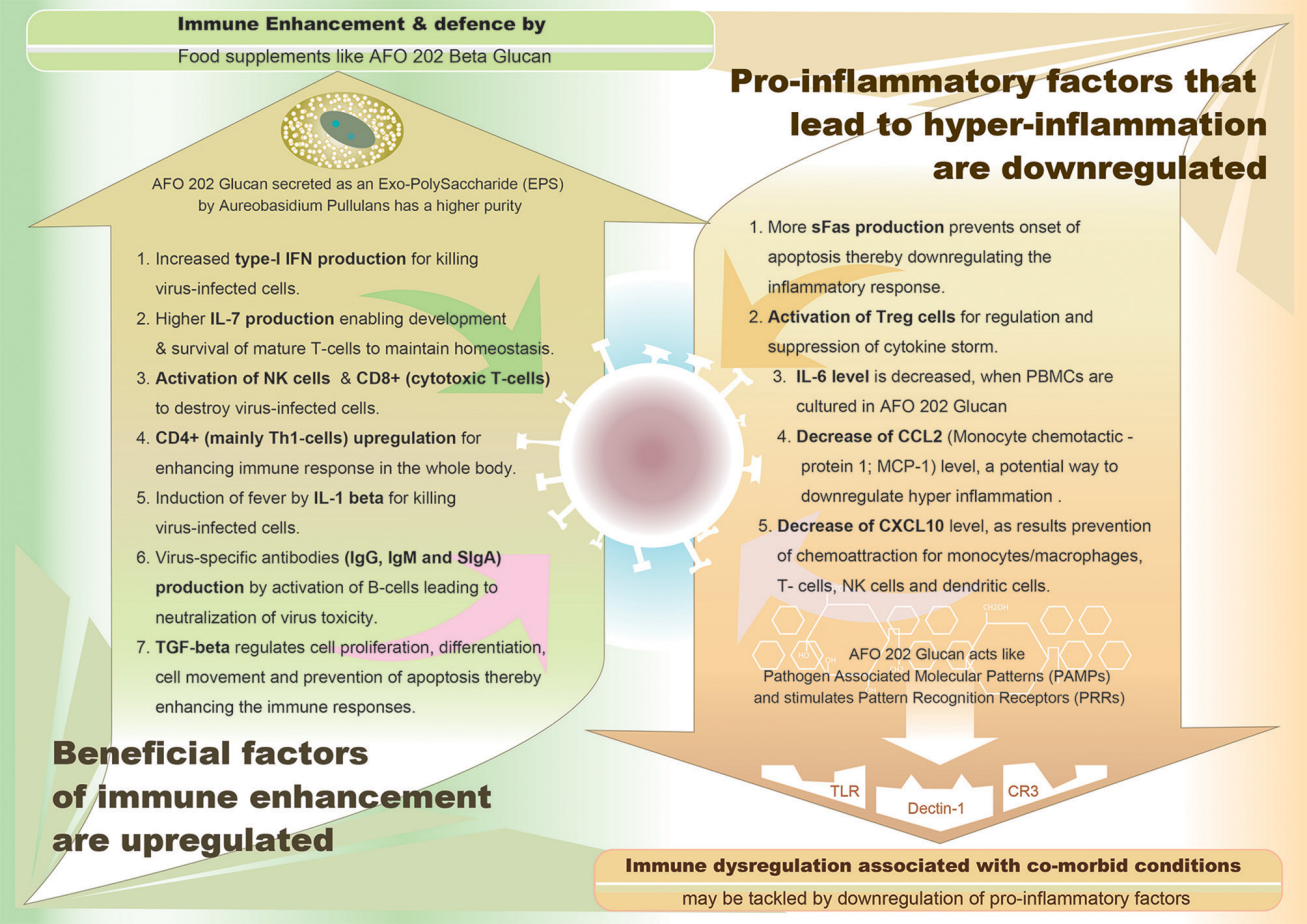
Graphical representation of demarcating between the upregulated immune enhancement factors and downregulated pro-inflammatory factors, both being beneficial effects of AFO-202 1-3,1-6 beta glucan, a biological response modifier (BRM).

Thus, in summary, in case of inflammatory pathway leading to cytokine storm, pro-inflammatory factors such as IL-6, IL-8, IL-1β, and GM-CSF and chemokines such as CCL2, CCL-5, IP-10, and CCL3, together with reactive oxygen species have been attributed to cause acute respiratory distress syndrome (ARDS) leading to pulmonary fibrosis and death. In COVID-19, high levels of serum pro-inflammatory cytokines (IFN-γ, IL-1, IL-6, IL-12, and TGFβ) and chemokines (CCL2, CXCL10, CXCL9, and IL-8) have been reported to be detected in cases with severe disease compared to patients with uncomplicated SARS (11). Also, continuous high levels of three cytokines (CXCL10, CCL7, and IL-1 receptor antagonist) has been associated with increased viral load, loss of lung function, lung injury, and a fatal outcome (12). While suppression of this pro-inflammatory cytokine storm is considered essential to combat COVID-19, some cytokines such as Type-I interferon and IL-7 have been found to be beneficial. Several studies are being conducted to study the effectiveness of IFN-α and IFN-β as drugs against SARS-CoV-2. Since lymphopenia and lymphocyte exhaustion are hallmarks of COVID-19, IL-7—the major cytokine promoting lymphocyte expansion and possibly reversal of T cell exhaustion is considered to be restoring immune system homeostasis. Intriguingly, in sera of patients with either mild or moderate or even severe forms of COVID-19, IL-2, and IL-7, the cytokines responsible for expansion and differentiation of various T cell subsets are increased which could be speculated as an attempt by the immune system to reverse lymphopenia and T cell exhaustion (13). A synopsis of pro-inflammatory cytokines which have to be down-regulated and the beneficial immune-factors which have to be enhanced to combat the COVID-19 are illustrated in Figure 1.

## Immune System Implications of Cardiovascular Diseases and COVID-19

The CFR of 2.3% in China was found to be elevated to 6.0% for patients with hypertension, 7.3% for patients with diabetes, and 10.5% for patience with CVD (14). Multiple studies have reported that patients with underlying cardiovascular comorbidities are at higher risk of severe COVID-19 infection that requires ICU care and of having complications like acute respiratory distress syndrome (ARDS), which may result in death. The mechanism has been attributed to reduced or impaired cardiovascular functional reserves in such CVD patients, which is worsened by the myocardial infarction precipitated by COVID-19, leading to increase myocardial demand, the worsening of ischemia and necrosis or, an increase in metabolic demand, which leads to heart failure and death. COVID-19 infection indirectly causes cardiac injury due to an overwhelming immune inflammatory response and cytokine storm. Other proposed mechanisms include SARS-CoV-2 viral invasion and direct damage of cardiomyocytes, as well as myocardial injury arising from severe hypoxia due to acute respiratory damage and also from another important process concerning angiotensin-converting enzyme 2 (ACE2) which is expressed in the heart and which SARS-CoV-2 uses as a receptor for entry into the cell (15). Among the cytokines that are elevated in the cytokine storm in COVID-19, IL-6 is important because it is the most strongly associated cytokine with coronary heart disease (CHD) (16). Interleukin-1b (IL-1b), tumor necrosis factor (TNF), and IL-17 have also been reported to be effective targets that can reduce cardiovascular progression (16).

## Immune System Implications of Diabetes and COVID-19

According to Yang et al. (17), among those studied who died due to COVID-19, 22% had cerebrovascular diseases and 22% had diabetes. A study of 1,099 patients with confirmed COVID-19 showed that among 173 who had severe disease, 23.7% had comorbid hypertension, 16.2% also had diabetes mellitus, 5.8% also had coronary heart disease, and 2.3% also had cerebrovascular disease. In another study among 140 patients who were admitted to the hospital for COVID-19, 30% had hypertension, and 12% had diabetes (18). The mechanisms proposed for increased susceptibility of patients with diabetes to COVID-19 include “(1) higher affinity cellular binding and efficient virus entry, (2) decreased viral clearance, (3) diminished T cell function, (4) increased susceptibility to hyperinflammation and cytokine storm syndrome, and (5) presence of CVD” (19). When the cytokine profile in diabetes was analyzed in relation to COVID-19, the focus was again on IL-6, which was reported to play a more deleterious role in COVID-19 infection (20).

## Immune System Implications of Chronic Kidney Disease and COVID-19

Chronic kidney disease, particularly patients with end-stage renal disease (ESRD) who are dependent on dialysis, are also in the high-risk category of acquiring severe disease and mortality due to COVID-19. When the underlying immune profile was analyzed, it was observed that cytokines such as interleukin-1 beta (IL-1 beta), tumor necrosis factor-alpha (TNF-alpha), and IL-6 induce an inflammatory state, playing a significant role in dialysis-related morbidity (21), again pointing to IL-6. In another report, between 30 and 50% of hemodialysis patients had elevated serum levels of inflammatory markers such as C-reactive protein and IL-6 (22). Furthermore, CKD has been associated with increases in immune senescence (23) and inflammation biomarkers (24).

## Immune System Implications of Cancer and Other Forms of Immunosuppression in COVID-19

Among 1,590 cases with confirmed COVID-19, Liang et al. found that 18 patients had a history of cancer. They concluded that patients with cancer had a higher risk of COVID-19 and poorer prognoses than those without cancer (25). While overwhelming inflammation and cytokine-associated lung injury are associated with the severity of COVID-19 in cancer patients according to Liang et al. (26) and Xia et al. (27) pointed out that the blunted immune status, characterized by overexpressed immunosuppressive cytokines, suppressed induction of proinflammatory danger signals, impaired dendritic cell maturation, and enhanced functional immunosuppressive leukocyte populations, could be the actual underlying factors exacerbating the severity of COVID-19 in cancer patients (26). Importantly, immunocompromised patients often present atypical presentations of viral diseases such as COVID due to the altered nature of the immune system (27). Among 10 kidney transplant recipients on immunosuppression who tested positive for SARS-CoV-2 by PCR, nine were admitted as in-patients, three patients (30%) died, and five (50%) developed acute kidney injury (28). A review of 89 studies on immune-suppressing or -stimulating drugs showed no conclusive evidence on the benefits of cytotoxic chemotherapy against COVID-19 infection (yet such benefits were observed during *in vitro* studies) or against the use of non-steroidal anti-inflammatory drugs (NSAIDs) and TNFα blockades. While clear evidence existed of an association between IL-6 peak levels and the severity of pulmonary complications, no evidence showed a beneficial impact of IL-6 inhibitors on modulating COVID-19 (29). Thus, immunosuppression has been reported to help with COVID-19, in lieu of the cytokine storm, and a kidney-transplanted patient infected with SARS CoV2 manifested only mild disease (30), thus adding more prominence to the fact that hyper-inflammation is the mechanism underlying COVID-19 progression. This is a major research area open for future perspectives.

## Immune Strategies for Combatting COVID-19

Having outlined the immune system characteristics in COVID-19 especially with co-morbidities, let us focus on the strategies for equipping the immune system to fight COVID-19. Innate immune response is highly critical for fighting viral infections and is decidedly dependent on interferon (IFN) type I responses, whose downstream cascade controls viral infection along with the induction of effective adaptive immune response. Innate immune cells recognize a virus's invasion through pathogen-associated molecular patterns (PAMPs), in the form of viral genomic RNA or the intermediates during viral replication, including dsRNA (30). This recognition event leads to the downstream signaling cascade being activated, which culminates in the expression of type I IFN and other pro-inflammatory cytokines. This initial response comprises the first line defense against viral infection at the entry site. For SARS-CoV and MERS-CoV, the response to viral infection by type I IFN is actually suppressed, which is closely associated with the disease's severity. SARS-CoV-2 also utilizes similar strategies of dampening type I IFN response. Furthermore, dysregulated type I IFN and inflammatory monocyte-macrophage influxes are the main cause of lethal pneumonia. Hence, the proposed immune combat strategies for COVID-19 involve suppressing of the cytokine storm by employing antagonists of some key pro-inflammatory cytokines, increasing the beneficial cytokines such as IL7, Type I IFN, along with treatment using anti-viral agents (30). While innate immune system–based strategies are key to therapeutics, an adaptive immune system holds the key to vaccine development (31). We postulate that a simpler and more effective approach will be nutritional intervention.

## Preventive and Therapeutic Nutritional Interventions for COVID-19

Supplementation of Vitamins A and D enhances immune response to influenza virus vaccination (32). Although Vitamin C is widely believed to help prevent viral infections, especially the common cold, a literature review of 640 studies failed to identify any conclusive evidence for Vitamin C prophylaxis in preventing the common cold (33, 34). Supplementation with micronutrients has mixed results. One RCT on 725 institutionalized elderly patients showed that low-dose supplementation of zinc together with selenium enhanced the humoral response after vaccination, in comparison to the control group (34), while in another RCT, neither daily multivitamin-mineral supplementation nor vitamin E (200 mg/day) showed a favorable effect on the incidence and severity of acute respiratory tract infections in well-nourished non-institutionalized elderly participants (35).

Nutraceuticals provide relief to people infected with encapsulated RNA viruses, such as influenza and coronavirus, by boosting their immune responses.

Beta-glucans are naturally occurring polysaccharides obtained from different sources such as oats, barley, bacteria, yeast, algae, and mushrooms. Beta-glucans derived from different sources have variation in their structure which is responsible for their specific biological properties (36). There have been nearly 7,000 publications reporting the immune-modulating effects of β-glucans (37). The immuno-modulating properties depend on the primary chemical structure of the β-glucans. β-glucans derived from fungi and yeast consisting of a (1,3)-β-linked backbone with small numbers of (1,6)-β-linked side chains, are known specifically for their immune-modulating effects (37). Vetvicka and Vetvickova have published several studies (38–40) comparing the immunological properties of different commercially available Beta Glucans in terms of effects on phagocytosis, IL-2 production, antibody secretion, Superoxide production, IFNγ production and inhibition of experimental cancer models, with their studies concluding that (i) glucans in general have strong stimulating effects on most aspects of the immune system; (ii) There are significant differences among tested glucans; (iii) highly purified and highly active glucans have strong and pleotropic effects stimulating all facets of immunological reactions while poorly defined glucans have only medium (if any) biological effects. Beta glucans such as the pleuran from the mushroom Pleurotus ostreatus has been reported to reduce the incidence of upper respiratory tract infection (URTI) symptoms and could increase the number of circulating NK cells (41). Therefore, beta glucans could be vital tools to fight against COVID-19 through the immune system.

We herein focus our discussion on a specific beta glucan: a 1-3,1-6 beta glucan from a black yeast called *Aureobasidium pullulans* AFO-202 strain (42, 43). This 1-3,1-6 beta glucan is secreted extra-cellularly by *Aureobasidium pullulans* and is collected from the culture medium, without the need for additional purification (44). Several studies have reported beta glucan to be a powerful immune stimulator that can activate macrophages and have positive immune actions on B-lymphocytes, natural killer cells, and suppressor T cells in the immune system (45–47). These actions are not direct but rather due to beta glucan being a biological response modifier (BRM) to enhance immunity (44). This AFO-202 beta glucan is also a biological response–modifier glucan (BRMG) whose biological response modifier (BRM) properties are significantly high (44) due to it being an exopolysaccharide without additional purification steps, which may hamper this nature. As indicated by Vetvicka and Vetvickova in their conclusions (38–40), since the AFO-202 β-1,3-1,6-glucan is highly pure and active, it exerts significant immunological actions. This AFO-202 beta glucan is recognized by the immune system as a PAMP equivalent and hence exerts immunological actions. This AFO-202 β-1,3-1,6-glucan is a soluble beta glucan that contains both high and low molecular weight beta-glucan. High molecular beta-glucan (H-BG) component has been found to stimulate the proliferation of lymphocytes with stronger effects. On the other hand, low molecular beta-glucan (L-BG) component reduces the levels of the inflammatory biomarkers (majorly cytokines), stimulates the cytokine and activates chemokine signaling pathways. In addition, L-BG effectively binds to dectin-1 (β-glucan receptor), and possesses antagonistic actions such as reactive oxygen production and cytokine synthesis from various immune cells such as macrophages, dendritic cells and endothelial cell etc. Since this AFO-202 beta-glucan contains both H-BG and L-BG it possesses ability to regulate whole of immune response for biological homeostasis (44). Beta-1,3/1,6-glucan derived from yeast has been listed by the US-FDA under the generally recognized as safe (GRAS) category (48). This AFO-202 Beta Glucan has been subjected to genotoxicity test, single oral administration test, 28-day or 90-day repeated dose study, long-term oral administration test (1 year) and has been certified to be safe (49). Also, this AFO-202 beta glucan has been approved by the Japanese Ministry of Health and available as a commercial food supplement for human consumption since 1996 (44).

Dectin-1 is a type II transmembrane receptor and the main beta glucan receptor involved in innate and adaptive immune responses to foreign antigens and pathogens; it is also the receptor for beta glucan as an immune function modulator (44). Dectin-1 cooperates with pattern-recognition receptors (PRRs) and Toll-like receptors (TLRs) in the innate immune responses to beta glucan recognition. Ikewaki et al. reported that this AFO-202 beta glucan induces the production of IL-8 and sFas through cultured peripheral blood mononuclear cells (PBMCs) and U937 cells but does not stimulate the production of IL-1β, IL-6, IL-12 (p70+40), IFN-γ, or TNF-α and actually decreases IL-6 levels (44). The enhancement of immune responses by AFO-202 β-1,3-1,6-glucan is associated with multiple signal transduction pathways involving several phosphoenzymes such protein kinase C (PKC), protein kinase A (PKA) inhibitor H-89 and protein tyrosine kinase (PTK) via intracellular mechanism(s). AFO- 202 beta glucan was shown to induce DNA synthesis (cell proliferation) in PBMCs via Dectin-1, CD11a CD54 (intercellular adhesion molecule-1; ICAM-1), HLA-class II, TLR-2 and TLR-4 and also induces the production of sFas. AFO-202 beta glucan stimulated U937 cells (a human monocyte-like cell line) inducing the production of sFas via Dectin-1, but not TLR-2 or TLR-4. The production of sFas by this beta glucan can prevent the onset of apoptosis, which is regulated by the Fas/FasL system, and can potentially downregulate inflammatory responses (44). When explored, beta glucan in one-way human mixed lymphocyte reaction (MLR) assay systems could activate suppressor cells—in particular, regulatory T cells (Treg)—and also induce the production of suppressive cytokines (44) which will be helpful in suppressing the cytokine storm observed in COVID-19. While the immunological actions of the AFO-202 beta glucan are evident and will have potential use against COVID-19 infection by immunosuppressing pro-inflammatory cytokines, several studies have also reported that this beta glucan can enhance immunity by increasing the levels of cytotoxic cells such as NK cells and macrophages, which will be the actual line of defense against the viruses. NK cell activity was significantly increased by this beta glucan in patients with *Leishmania amazonensis* infection (50). This beta glucan had regulatory or enhancing properties on poultry non-specific cellular immunity in a study on Peking ducks (51) and may enhance the immune response to avian influenza A H5 vaccine (52). This AFO-202 beta glucan increased the NK cell and macrophage counts in cancer patients and elderly patients (53). Glucan supplementation enhanced the immune response against an influenza challenge in mice (54). In a study analyzing the efficacy of this AFO-202 beta glucan in protecting mice infected with a lethal titer of the A/Puerto Rico/8/34 (PR8; H1N1) strain of influenza virus, the survival rate was significantly increased by the beta glucan's administration after a sublethal infection of PR8 virus, and pre-treatment with beta glucan significantly repressed the replication of the PR8 virus (55). Yeast (1,3)-(1,6)-beta-glucan also reduced the severity of upper respiratory tract infections in a double-blind, randomized, placebo-controlled study (56).

## AFO-202 Beta Glucan and Relevance to COVID-19 Patients With Co-Morbidities

While AFO-202 beta glucan supplementation can be a potential strategy to fight COVID-19 infection due to its immune-enhancing activity in terms of IFN-γ-increasing capability (44), whose suppression is characteristic of SARS-COv2 infection (31), its consumption should be emphasized for people with comorbidities. IL-6 is the most commonly elevated cytokine in cytokine storms from conditions involving chronic micro-inflammation, such as CVD, diabetes and CKD (12, 13, 17, 18). This AFO-202 beta glucan decreases IL-6 levels (44). The increase in sFAS, which helps in regulating the immune response by immune suppression, will be highly valuable in regulating the cytokine storms and hyper-inflammation associated with COVID-19 (44). In regard to the pro-inflammatory and beneficial cytokines listed in the introduction section of the manuscript (11, 13), AFO-202 beta glucan through IL8 causes activation, migration and chemotaxis of neutrophils for killing virus-infected cells. This beta glucan also causes decrease of CCL2 (Monocyte chemotactic protein 1; MCP-1) and decrease of CXCL10 levels, as result of which there will be prevention of chemoattraction for monocytes/macrophages, T-cells, NK cells, and dendritic cells thereby suppressing immune response. In addition, promotion of T-cell adhesion to endothelial cells and anti-tumor activity accompanied by enhancement of immune responses occur. Increase of type-I IFN production by AFO-202 beta glucan helps in killing virus-infected cells (44). Further, increase of IL-7 production leads to development and survival of mature T-cells to maintain homeostasis. Activation of CD8+ (cytotoxic T-cells) helps in anti-viral immunity while activation of CD4+ (mainly Th1-cells) and Treg cells helps in regulatory immune response and suppression of cytokine storm with severe inflammation. Activation of B-cells results in production of virus-specific antibodies (IgG, IgM, and sIgA) for neutralization of virus toxicity (44). Figure 1 illustrates the beneficial effects of the AFO-202 beta glucan by upregulating the immune enhancement factors and downregulating the pro-inflammatory factors, in COVID-19 in a nutshell.

The regulatory immune profile enhanced by AFO-202 beta glucan (35) will assist with immune modulation in patients with cancer. For kidney transplant recipients and patients with immune suppression, the NK cell– and macrophage-enhancing activity will come to play in antiviral immunity (44) thereby helping them to fight COVID-19.

Another interesting aspect is that gut microbiota can influence the generation of innate memory and the functional reprogramming of bone marrow progenitors, which can help to protect against infections (57). Their dysbiosis also leads to variety of immune-mediated inflammatory disorders (57). Beta glucan's beneficial effects in reducing diseases like CVD have also been attributed to their action influencing gut microbiota (58), and the immune-modulation of beta glucans by acting on gut microbiota can help to alleviate an inflammatory immune profile (59), thereby leading to beneficial effects. These advantages mean that the beta glucan should more importance in fighting COVID-19, specifically in the presence of chronic inflammation–associated comorbidities. Thus, for those at high risk who suffer from a wide range of comorbidities, consumption of this food supplement—whose safety has been proven by consumption over two decades (44, 48, 49)—will be a prospective preventive option and even as a supportive choice during therapeutics in the fight against this deadly COVID-19 pandemic.

## Limitations in Considering AFO-202 Beta Glucan Type of Nutritional Supplements in COVID-19

Beta glucans in general are considered to have immune modulatory effect which differs based on their source and structure of each type of beta glucan; the AFO-202 beta glucan due to its purity and other unique characteristics, is considered relatively advantageous in terms of immune enhancement and immuno-modulatory effects to be strategically efficient in tackling COVID-19 implications. Though, the AFO-202 beta glucan has not yet been subjected to a clinical study in COVID-19 positive patients, which is a limitation, before such a study, variabilities among patients with COVID-19 due to inherent factors or acquired variations or individual factors such as co-morbid conditions will have to be considered to evaluate the efficacy of AFO-202 beta glucan. Because, inherent factors such as genetic variation in terms of human leukocyte antigen (HLA) polymorphisms (60), mutations in ACE-2 gene (61), age, general health, and nutrition (62), acquired variations such as cross immunity provided by vaccines like BCG vaccine (63) and Japanese encephalitis (JE) vaccine (64) and individual variations due to co-morbidities which cause dysregulation, described in this manuscript, have been reported to influence the variation in susceptibility of individuals to COVID-19. Another limitation is on the exact stage of pathophysiology until which the immune-enhancement or immune-modulation mechanisms could play a role in tackling COVID-19 and whether those mechanisms will come into play when hyper-inflammation leads to a cytokine storm setting in, causing respiratory illness and multi-organ dysfunction (65). Recently, thrombo-embolic complications culminating in micro-or macrovascular stroke have been reported in some patients with mild COVID-19 infection (66). Increased levels of IL6 leading to secretion of vascular endothelial growth factor (VEGF) and reduction of E-cadherin expression contributing to vascular permeability by which SARS-CoV-2 invades endothelial cells inducing an endothelial inflammation has been cited as the probable mechanism behind (67, 68). Thus, further in-depth studies are warranted to evaluate the effects of nutritional supplements such as the AFO-202 beta glucan in such implications of different interacting pathways of the immune system leading to thrombo-embolic manifestations as well.

## Conclusion

The immune system is a double-edged sword (69) that has balance between its primary activity of defense against foreign pathogens, oncogenesis, and circulating cancer cells while maintaining their limitations from overacting and ending up in a hyperinflammatory status, which leads to severe cytokine storm in COVID-19 patients. Although specific targeted molecules and agents that can act on each step of such an immune system may be efficacious for providing beneficial effects they continue to have adverse reactions. Given this background, broadly acting non-harmful strategies are currently considered essential, given that the lack of a definitive vaccine to “complex” the COVID-19 pandemic is threatening vulnerable populations with immune dysregulation. Through this analysis, we have found that a proven primary immune defense–improving and immune-modulation oriented nutritional supplement such as the AFO-202 beta glucan could be tried in these patients in a multi-centric study to prove its efficacy. Its consumption as a food supplement has proven safe for more than two decades.

## Author Contributions

NI, K-SR, SK, and SA contributed to conception and design of the study. RS performed the literature search and data analysis. SA and SP drafted the manuscript. NI, K-SR, VS, and SA performed critical revision of the manuscript. All authors contributed to manuscript revision, read, and approved the submitted version.

## Conflict of Interest

K-SR is an employee of INDICASAT AIP, Panama. NI is an employee of Kyushu University of Health and Welfare, Institute of Immunology, Junsei Educational Institute, Nobeoka, Miyazaki, Japan. VS is an employee of Yashoda Hospital, India. RS and SP are employees of NCRM, India. SK is a faculty of EELS & employee of Edogawa Hospital, Japan. SA is a faculty in Yamanashi University, EELS, Edogawa Hospital, Japan & NCRM, India, as well as shareholder in GN Corporation, Japan which in turn is a shareholder in the manufacturing company of the AFO-202 Beta Glucan.

## References

[1] Wang C Horby PW Hayden FG Gao GF. A novel coronavirus outbreak of global health concern. Lancet. (2020) 395:470–3. 10.1016/S0140-67362030185-9 31986257 PMC7135038

[2] Lu R Zhao X Li J Niu P Yang B Wu H. Genomic characterisation and epidemiology of 2019 novel coronavirus: implications for virus origins and receptor binding. Lancet. (2020) 395:565–74. 10.1016/S0140-67362030251-8 32007145 PMC7159086

[3] World Health Organization Coronavirus (COVID-19). (2020). Available online at: https://covid19.who.int/ (accessed May 5, 2020).

[4] Chakraborty C Sharma AR Sharma G Bhattacharya M Lee SS. SARS-CoV-2 causing pneumonia-associated respiratory disorder (COVID-19): diagnostic and proposed therapeutic options. Eur Rev Med Pharmacol Sci. (2020) 24:4016–26. 10.26355/eurrev_202004_20871 32329877

[5] Centre for Evidence-Based Medicine Global Covid-19 Case Fatality Rates. (2020). Available online at: https://www.cebm.net/covid-19/global-covid-19-case-fatality-rates/ (accessed May 5, 2020).

[6] Singh AK Gupta R Misra A Comorbidities in COVID-19: Outcomes in hypertensive cohort and controversies with renin angiotensin system blockers. Diabetes Metab Syndr. (2020) 14:283–7. 10.1016/j.dsx.2020.03.016 32283499 PMC7144598

[7] Pedersen F Ho YC. SARS-CoV-2: a storm is raging. J Clin Invest. (2020) 130:2202–5. 10.1172/JCI137647 32217834 PMC7190904

[8] Li G Fan Y Lai Y Han T Li Z Zhou P Coronavirus infections and immune responses. J Med Virol. (2020) 92:424–32. 10.1002/jmv.25685 31981224 PMC7166547

[9] Cao X. COVID-19: immunopathology and its implications for therapy. Nat Rev Immunol. (2020) 20:269–70. 10.1038/s41577-020-0308-3 32273594 PMC7143200

[10] Lagunas-Rangel FA. Neutrophil-to-lymphocyte ratio and lymphocyte-to- C-reactive protein ratio in patients with severe coronavirus disease 2019 (COVID-19): A meta-analysis. J Med Virol. (2020) 25819. 10.1002/jmv. 32242950 PMC7228336

[11] Sun X Wang T Cai D Hu Z Chen J Liao H. Cytokine storm intervention in the early stages of COVID-19 pneumonia. Cytokine Growth Factor Rev. (2020) 53:38–42. 10.1016/j.cytogfr.2020.04.002 32360420 PMC7182527

[12] Vaninov N. In the eye of the COVID-19 cytokine storm. Nat Rev Immunol. (2020) 20:277. 10.1038/s41577-020-0305-6 32249847 PMC7132547

[13] Jamilloux Y Henry T Belot A Viel S Fauter M El Jammal T Should we stimulate or suppress immune responses in COVID-19? Cytokine and anti-cytokine interventions. Autoimmun Rev. (2020) 19:102567 10.1016/j.autrev.2020.102567 32376392 PMC7196557

[14] Perricone C Triggianese P Bartoloni E Cafaro G Bonifacio AF Bursi R. The anti-viral facet of anti-rheumatic drugs: lessons from COVID-19. J Autoimmun. (2020) 111:102468. 10.1016/j.jaut.2020.102468 32317220 PMC7164894

[15] Tan W Aboulhosn J. The cardiovascular burden of coronavirus disease 2019 (COVID-19) with a focus on congenital heart disease. Int J Cardiol. (2020) 309:70–7. 10.1016/j.ijcard.2020.03.063 32248966 PMC7102656

[16] Clarke R Valdes-Marquez E Hill M Gordon J Farrall M Hamsten A. Plasma cytokines and risk of coronary heart disease in the PROCARDIS study. Open Heart. (2018) 5:e000807. 10.1136/openhrt-2018-000807 29713486 PMC5922567

[17] Williams JW Huang LH Randolph GJ. Cytokine circuits in cardiovascular disease. Immunity. (2019) 50:941–54. 10.1016/j.immuni.2019.03.007 30995508 PMC6924925

[18] Fang L Karakiulakis G Roth M. Are patients with hypertension and diabetes mellitus at increased risk for COVID-19 infection? Lancet Respir Med. (2020) 8:e21. 10.1016/S2213-2600(20)30116-8 32171062 PMC7118626

[19] Muniyappa R Gubbi S. COVID-19 pandemic, coronaviruses, and diabetes mellitus. Am J Physiol Endocrinol Metab. (2020) 318:E736–41. 10.1152/ajpendo.00124.2020 32228322 PMC7191633

[20] Maddaloni E Buzzetti R. Covid-19 and diabetes mellitus: unveiling the interaction of two pandemics. Diabetes Metab Res Rev. (2020) e33213321. 10.1002/dmrr.3321 32233018 PMC7228318

[21] Ferrey AJ Choi G Hanna RM Chang Y Tantisattamo E Ivaturi K. A case of novel coronavirus disease 19 in a chronic hemodialysis patient presenting with gastroenteritis and developing severe pulmonary disease. Am J Nephrol. (2020) 51:337–342. 10.1159/000507417 32222713 PMC7179539

[22] Pertosa G Grandaliano G Gesualdo L Schena FP. Clinical relevance of cytokine production in hemodialysis. Kidney Int Suppl. (2000) 76:S104–11 10.1046/j.1523-1755.2000.07613.x 10936806

[23] Jofré R Rodriguez-Benitez P López-Gómez JM Pérez-Garcia R. Inflammatory syndrome in patients on hemodialysis. J Am Soc Nephrol. (2006) 17:S274–80. 10.1681/ASN.2006080926 17130274

[24] Crépin T Legendre M Carron C Vachey C Courivaud C Rebibou JM. Uraemia-induced immune senescence and clinical outcomes in chronic kidney disease patients. Nephrol Dial Transplant. (2020) 35:624–32. 10.1093/ndt/gfy276 30202981

[25] Liang W Guan W Chen R Wang W Li J Xu K. Cancer patients in SARS-CoV-2 infection: a nationwide analysis in China. Lancet Oncol. (2020) 21:335–7. 10.1016/S1470-2045(20)30096-6 32066541 PMC7159000

[26] Xia Y Jin R Zhao J Li W Shen H. Risk of COVID-19 for patients with cancer. Lancet. Oncol. (2020). 21:e180. 10.1016/S1470-2045(20)30150-9 32142622 PMC7130057

[27] Guillen E Pineiro GJ Revuelta I Rodriguez D Bodro M Moreno A. Case report of COVID-19 in a kidney transplant recipient: does immunosuppression alter the clinical presentation? Am J Transplant. (2020). 10.1111/ajt.15874 32198834 PMC7228209

[28] Nair V Jandovitz N Hirsch JS. Nair G Abate M Bhaskaran M COVID-19 in kidney transplant recipients. Am J Transplant. (2020). 10.1111/ajt.15967 PMC7267603 32351040

[29] Russell B Moss C George G Santaolalla A Cope A Papa S. Associations between immune-suppressive and stimulating drugs and novel COVID-19-a systematic review of current evidence. Ecancermedicalscience. (2020) 14:1022. 10.3332/ecancer.2020.1022 32256705 PMC7105343

[30] Seminari E Colaneri M Sambo M Gallazzi I Di Matteo A Silvia R. SARS Cov2 infection in a renal transplanted patients. A case report. Am J Transplant. (2020). 10.1111/ajt.15902 32243672 PMC9800469

[31] Prompetchara E Ketloy C Palaga T. Immune responses in COVID-19 and potential vaccines: lessons learned from SARS and MERS epidemic. Asian Pac J Allergy Immunol. (2020) 38:1–9. 10.12932/AP-200220-0772 32105090

[32] Patel N Penkert RR Jones BG Sealy RE Surman SL Sun Y. Baseline serum vitamin A and D levels determine benefit of oral vitamin A&D supplements to humoral immune responses following pediatric influenza vaccination. Viruses. (2019) 11:907. 10.3390/v11100907 31575021 PMC6832482

[33] Jayawardena R Sooriyaarachchi P Chourdakis M Jeewandara C Ranasinghe P Enhancing immunity in viral infections, with special emphasis on COVID-19: a review. Diabetes Metab Syndr. (2020) 14:367–82. 10.1016/j.dsx.2020.04.015 32334392 PMC7161532

[34] Girodon F. Impact of trace elements and vitamin supplementation on immunity and infections in institutionalized elderly patients: a randomized controlled trial. Arch Intern Med. (1999) 159:748–54. 10.1001/archinte.159.7.748 10218756

[35] Graat JM Schouten EG Kok FJ. Effect of daily vitamin E and multivitamin-mineral supplementation on acute respiratory tract infections in elderly Persons-A randomized controlled trial. JAMA. (2002) 288:715–21. 10.1001/jama.288.6.715 12169075

[36] Akramiene D Kondrotas A Didziapetriene J Kevelaitis E. Effects of beta-glucans on the immune system. Medicina. (2007) 43:597–606. 10.3390/medicina43080076 17895634

[37] Stier H Ebbeskotte V Gruenwald J. Immune-modulatory effects of dietary Yeast Beta-1,3/1,6-D-glucan. Nutr. J. (2014) 13:38. 10.1186/1475-2891-13-38 24774968 PMC4012169

[38] Vetvicka V Vetvickova J Comparison of immunological effects of commercially available β-glucans. Appl Sci Rep. (2014) 1:2 10.7243/2054-9903-1-2

[39] Vetvicka V Vetvickova J. Comparison of immunological effects of commercially available beta-glucans: part III. Int Clin Pathol J. (2016) 2:78–83. 10.15406/icpjl.2016.02.0004629491056

[40] Vetvicka V Vetvickova J. Glucans and cancer: comparison of commercially available β-glucans - Part IV. Anticancer Res. (2018) 38:1327–33. 10.21873/anticanres.12355 29491056

[41] Bergendiova K Tibenska E Majtan J. Pleuran (β-glucan from Pleurotus ostreatus) supplementation, cellular immune response and respiratory tract infections in athletes. Eur J Appl Physiol. (2011) 111:2033–40. 10.1007/s00421-011-1837-z 21249381

[42] Dedeepiya VD Sivaraman G Venkatesh AP Preethy S Abraham SJ. Potential effects of nichi glucan as a food supplement for diabetes mellitus and hyperlipidemia: preliminary findings from the study on three patients from India. Case Rep Med. (2012) 2012:895370. 10.1155/2012/895370 23304164 PMC3529881

[43] Ganesh JS Rao YY Ravikumar R Jayakrishnan GA Iwasaki M Preethy S. Beneficial effects of black yeast derived 1-3, 1-6 Beta Glucan-Nichi Glucan in a dyslipidemic individual of Indian origin–a case report. J Diet Suppl. (2014) 11:1–6. 10.3109/19390211.2013.859211 24409973

[44] Ikewaki N Fujii N Onaka T Ikewaki S Inoko H. Immunological actions of Sophy beta-glucan (beta-1,3-1,6 glucan), currently available commercially as a health food supplement. Microbiol Immunol. (2007) 51:861–73. 10.1111/j.1348-0421.2007.tb03982.x 17895603

[45] Brown GD Gordon S. Fungal beta-glucans and mammalian immunity. Immunity. (2003) 19:311–5. 10.1016/S1074-7613(03)00233-4 14499107

[46] Cramer DE Allendorf DJ Baran JT Hansen R Marroquin J Li B. Beta-glucan enhances complement-mediated hematopoietic recovery after bone marrow injury. Blood. (2006) 107:835–40. 10.1182/blood-2005-07-2705 16179370 PMC1895628

[47] Ohno N Furukawa M Miura NN Adachi Y Motoi M Yadomae T. Antitumor beta glucan from the cultured fruit body of Agaricus blazei. Biol Pharm Bull. (2001) 24:820–8. 10.1248/bpb.24.820 11456124

[48] FDA GRAS Classification of Beta-1,3/1,6-Glucan or Beta-1,3(D)-Glucan. (2020). Available online at: https://www.betaglucan.org/fdagras/ (accessed June 6, 2020).

[49] Ikewaki N Sonoda T Miyazawa Y Onizuka S Chikamori M Serum levels of β-1,3-1,6 glucan-specific antibodies and immune biomarkers in normal individuals. J Kyushu Univ Health Welfare. (2018) 19:87–94.

[50] Yatawara L Wickramasinghe S Nagataki M Takamoto M Nomura H Ikeue Y. Aureobasidium-derived soluble branched (1,3-1,6) beta-glucan (Sophy beta-glucan) enhances natural killer activity in Leishmania amazonensis-infected mice. Korean J Parasitol. (2009) 47:345–51. 10.3347/kjp.2009.47.4.345 19967081 PMC2788712

[51] Tang XY Gao JS Yuan F Zhang WX Shao YJ Sakurai F. Effects of Sophy β-glucan on growth performance, carcass traits, meat composition, and immunological responses of Peking ducks. Poult Sci. (2011) 90:737–45. 10.3382/ps.2010-01008 21406357

[52] Le T Le T Doan TH Quyen D Le KX Pham V. The adjuvant effect of Sophy β-glucan to the antibody response in poultry immunized by the avian influenza A H5N1 and H5N2 vaccines. J Microbiol Biotechnol. (2011) 21:405–11. 10.4014/jmb.1011.11024 21532325

[53] Ikewaki N Results of oral consumption of AFO-202 Beta Glucan in elderly volunteers and cancer patients through NK cell activity. Abstract presented at 28th Annual Meeting of Japanese Society for Parenteral and Enteral Nutrition (2013).

[54] Vetvicka V Vetvickova J. Glucan supplementation enhances the immune response against an influenza challenge in mice. Ann Transl Med. (2015) 3:22. 10.3978/j.issn.2305-5839.2015.01.08 25738142 PMC4322159

[55] Muramatsu D Iwai A Aoki S Uchiyama H Kawata K Nakayama Y. β-Glucan derived from Aureobasidium pullulans is effective for the prevention of influenza in mice. PLoS ONE. (2012) 7:e41399. 10.1371/journal.pone.0041399 22844473 PMC3402398

[56] Dharsono T Rudnicka K Wilhelm M Schoen C. Effects of yeast (1,3)-(1,6)-beta-glucan on severity of upper respiratory tract infections: a double-blind, randomized, placebo-controlled study in healthy subjects. J Am Coll Nutr. (2019) 38:40–50. 10.1080/07315724.2018.1478339 30198828

[57] Elson CO Alexander KL. Host-microbiota interactions in the intestine. Dig Dis. (2015) 33:131–6. 10.1159/000369534 25925913

[58] Wang Y Ames NP Tun HM Tosh SM Jones PJ Khafipour E. High molecular weight barley β-glucan alters gut microbiota toward reduced cardiovascular disease risk. Front Microbiol. (2016) 7:129. 10.3389/fmicb.2016.00129 26904005 PMC4748052

[59] Raa J. Immune modulation by non-digestible and non-absorbable beta-1,3/1,6-glucan. Microb Ecol Health Dis. (2015) 26:27824. 10.3402/mehd.v26.27824 26031679 PMC4451094

[60] Nguyen A David JK Maden SK Wood MA Weeder BR Nellore A. Human leukocyte antigen susceptibility map for SARS-CoV-2. J Virol. (2020) JVI.00510–20. 10.1128/JVI.00510-20 32303592 PMC7307149

[61] Stawiski EW Diwanji D Suryamohan K Gupta R Fellouse FA Sathirapongsasuti JF Human ACE2 receptor polymorphisms predict SARS-CoV-2 susceptibility. bioRxiv. (2020) 2020.04.07.024752. 10.1101/2020.04.07.024752x PMC8041869 33846513

[62] Zabetakis I Lordan R Norton C Tsoupras A. COVID-19: the inflammation link and the role of nutrition in potential mitigation. Nutrients. (2020) 12:E1466. 10.3390/nu12051466 32438620 PMC7284818

[63] Rajarshi K Chatterjee A Ray S BCG vaccination strategy for preventaion against COVID-19: Hype or Hope? Preprints. (2020) 2020040351 10.20944/preprints202004.0351.v1 PMC7255206 32835211

[64] Katoh S Obayashi T Ganesh JS Iwasaki M Preethy S Abraham SJ. Cross-protection induced by encephalitis vaccines against COVID-19 might be a reason for relatively lower mortality rate in some Countries. Arch Acad Emerg Med. (2020) 8:e54. 10.22037/aaem.v8i1.683.g806 32440665 PMC7212070

[65] Cascella M Rajnik M Cuomo A Dulebohn SC Di Napoli R. Features, Evaluation and Treatment Coronavirus (COVID-19) [Updated 2020 May 18]. In: StatPearls [Internet]. Treasure Island (FL): StatPearls Publishing (2020) 32150360

[66] Fara MG Stein LK Skliut M Morgello S Fifi JT Dhamoon MS. Macrothrombosis and stroke in patients with mild Covid-19 infection. J Thromb Haemost. (2020). 10.1111/jth.14938 32464707 PMC7283879

[67] Steinack C Hage R Benden C Schuurmans MM SARS-CoV-2 and norovirus co-infection after lung transplantation. Transplantology. (2020) 1:16–23. 10.3390/transplantology1010002

[68] Tanaka T Narazaki M Kishimoto T. Immunotherapeutic implications of IL-6 blockade for cytokine storm. Immunotherapy. (2016) 8:959–70. 10.2217/imt-2016-0020 27381687

[69] Shahabi nezhad F Mosaddeghi P Negahdaripour M Dehghani Z Farahmandnejad M Moghadami M Therapeutic approaches for COVID-19 based on the dynamics of interferon-mediated immune responses. Preprints. (2020) 2020030206. 10.20944/preprints202003.0206.v1

